# Book Reviews

**Published:** 1898-04

**Authors:** 


Manual of Gynecology. By Henry T. Byford, Professor of Gynecology and Clinical Gynecology in the College of Physicians and Surgeons of Chicago; Professor of Gynecology and Clinical Gynecology in the Woman's Medical School of Northwestern University; Professor of Gynecology in the Post-Graduate Medical School of Chicago. Octavo, pp. xxiii.—596, containing 341 illustrations, many of which are original Philadelphia: P. Blakiston, Son & Co., 1012 Walnut street. 1897.

The demand for a second edition of this work so soon after the first was put forth is an assured indication of its popularity. When writing of the first edition (Volume XXXV., p. 761, April, 1896,) we stated that we regarded Byford's manual as one of the best published. With the improvements the author has made in this new edition we desire to accentuate our former opinion in a most forceful manner.

The author has retained the general arrangement, but has devoted a separate part each to carcinoma, sarcoma and cystic tumors. A new part also has been added on the anatomy of the pelvic organs. Angioma, hypertrophy and atrophy of the uterus have received separate mention and there are new chapters on venereal diseases. The chapters on hyperplasia, chronic inflammation and subinvolution of the uterus have been rewritten, while those on urinary and fecal fistula, and ectopic pregnancy, have been reconstructed.

New illustrations have been added and a system of marginal notes serves to facilitate reference to the text. It is by far the best student's manual in the field and exhibits the work of a master from introduction to index.

CLINICAL METHODS, being an Introduction to the Practical Study of Medicine. By ROBERT HUTCHISON, M. D., M. R. C. P., Demonstrator of Physiology in London Hospital Medical College, and HARRY RAINY, F. R. C. P. P., F. R. S. E., University Tutor in Clinical Medicine, Royal Infirmary, Edinburgh. Duodecimo, pp. 562; 137 engravings and eight colored plates. Philadelphia and New York: Lea Brothers & Co., Publishers.

An approach to perfection in clinical methods is most desirable, and any literature tending to facilitate approximation thereto is to be welcomed. The title of this little work is attractive from its very novelty. Its object is to direct closer attention to clinical investigation by methodising all the details of clinical work from case-taking to diagnosis.

A special chapter is assigned to the examination of children. This will be found of especial interest to every physician who has occasion to treat children. There are also chapters on the examination of pathological fluids and on clinical bacteriology.

The book is prepared by two authors who are thoroughly alive to their subject and who have created a most interesting work that is well illustrated and handsomely printed and bound.

---

ABBOTT'S BACTERIOLOGY. Fourth and revised edition. Just ready. The Principles of Bacteriology. A practical manual for students and physicians. By A. C. ABBOTT, M. D., Professor of Hygiene and Director of the Laboratory of Hygiene, University of Pennsylvania, Philadelphia. Fourth edition, enlarged and thoroughly revised. Handsome 12mo, 542 pages, 106 illustrations, of which nineteen are colored. Philadelphia and New York: Lea Brothers & Co., Publishers.

The previous editions of this excellent work have been noticed in the JOURNAL. The first in the issue for April, 1892, the second in January, 1895, and the third in August, 1896. In this edition the author attempts to include the more important of the newer suggestions relating to bacteriology and he has introduced illustrated descriptions of the bacillus of bubonic plague, of the bacillus of influenza and of the micrococcus of gonorrhea, and a number of other new illustrations which serve to still further elucidate the text. The appearance of new editions of this work at such short intervals indicates progression in the science of bacteriology and an endeavor on the part of the author to keep pace with it. We commend the book to every student and teacher of bacteriology.

The American Year-Book of Medicine and Surgery ; being a yearly digest of scientific progress and authoritative opinion in all branches of medicine and surgery, drawn from journals, monographs and text-books of the leading American and foreign authors and investigators. Collected and arranged with critical editorial comments by eminent American specialists and teachers. Under the general editorial charge of George M. Gould, M. D. One volume of nearly 1,200 pages, profusely illustrated with numerous woodcuts in text, and thirty-three half-tone and colored plates. Philadelphia : W. B. Saunders. 1898.

The third annual volume of this important compilation of medical literature is constructed on lines similar to the preceding volumes. The editor-in-chief and his staff have performed a great labor in grouping, abstracting and compiling such an enormous amount of literature and placing it between two covers, thus making it easily accessible as a work of reference.

We have, heretofore, expressed our opinion as to the scope and import of the year-book—an opinion that we wish to reiterate and still further accentuate here and now ; in other words, we feel that by and large this is the most comprehensive and valuable of the many year-books, annuals or digests offered to the medical profession. The publisher, too, is entitled to a meed of praise for his part in creating such a handsome volume.

---

A Handbook of Therapeutics. By Sidney Ringer, M. D., F. R. S. Holme Professor of Clinical Medicine, Universily College ; Physician to University College Hospital, and Harrington Sainsbury, M. D., F. R. C. P., Physician to the Royal Free Hospital, and the City of London Hospital for Diseases of the Chest, Victoria Park. Thirteenth edition. Octavo, pp. xi.—746. New York : William Wood & Company. 1897.

It is eight years since the twelfth edition of Ringer's therapeutics visited us, during which time many changes have taken place, not only in therapeutics, but also in materia medica. In Volume XXVIII., p. 625, May, 1889, we made somewhat extended comment upon the twelfth edition of this valuable treatise, which has become well-nigh classic, and to which we refer the reader for a general synopsis as well as an opinion of the work.

The make-up of the book is changed in this edition to small octavo, in place of standard octavo as heretofore. The amount of material, however, is rather increased than diminished, since this edition contains 746 pages against 524 in the twelfth. Owing to the long interval that has elapsed between this and the last edition, the list of new drugs as well as of new methods has been somewhat voluminous and to sift the tried from the untried, the useful from the worthless, the good from the bad, is no light tax upon the ingenuity, judgment, experience and skill of the most accomplished and practical author, yet all this has been most happily accomplished by Ringer.

Serum therapy is accorded a separate chapter and a short section upon the use of the digestive ferments appears in connection with the invalid dietary. The Nauheim-Schott treatment of cardiac failure is herein described in detail. It is important in these days of overwork and underrest, in which cardiac strain is so liable, for the practitioner of medicine to be amply equipped with every known method of prevention and cure of this disorder.

The thirteenth edition continues on the same constructed lines as those that have preceded it, holding high above every other principle that it is of the first importance for symptoms to receive weighty consideration, since in the majority of cases we are compelled to treat the secondary effects of a malady rather than the primary disease. In other words, it is Ringer's constant endeavor to make clinical considerations indicate or contraindicate the employment of remedies; hence the use of drugs in disease are dwelt upon rather than their physiological action, thus making the book a work of clinical therapeutics.

It is a treatise that should be in the hands of every teacher and practitioner of medicine. The publishers have made this the handsomest edition of the work yet issued.

---

INTERNATIONAL CLINICS. A Quarterly of Clinical Lectures on Medicine, Neurology, Surgery, Gynecology, Obstetrics, Ophthalmology, Laryngology, Pharyngology, Rhinology, Otology and Dermatology, and specially prepared articles on treatment. By professors and lecturers in the leading medical colleges of the United States, Germany, Austria, France, Great Britain and Canada. Edited by Judson Daland, M. D., (Univ. of Penna.) Philadelphia, Instructor in Clinical Medicine and Lecturer on Physical Diagnosis in the University of Pennsylvania; Assistant Physician to the Hospital of the University of Pennsylvania, etc.; Fellow of the College of Physicians of Philadelphia. J. Mitchell Bruce, M. D., F. R. C. P., London, England, Physician to, and Lecturer on, the Principles and Practice of Medicine at the Charing Cross Hospital. David W. Finlay, M. D., F. R. C. P., Aberdeen, Scotland, Professor of Practice of Medicine in the University of Aberdeen; Physician to, and Lecturer on, Clinical Medicine in the Aberdeen Royal Infirmary, etc. Volume IV. Seventh series. 1898. Octavo, pp, xii.—363. Philadelphia: J. B. Lippincott Co. 1897.

This volume is divided into the following-named departments: drugs and intermediate agents, treatment, medicine, neurology, surgery, gynecology and obstetrics, ophthalmology, laryngology and rhinology, and dermatology.

The principal contributors are Roberts Bartholow, Harrington Sainsbury, Hobart Amory Hare, D. D. Stewart, I. N. Love, A. A. Eshner, Charles Lyman Greene, John Madison Taylor, Edward D. Fisher, Joseph M. Mathews, James F. W. Ross, Charles Greene Cumston, Paul F. Mundé, Professor Ricard, of Paris, Thomas R. Pooley, William Cheatham and I. E. Atkinson.

The book is constructed on similar lines to its predecessors and is an interesting and useful addition to the physician's library.

---

Transactions of the American Surgical Association. Volume XV. Edited by DeForest Willard, A. M., M. D., Ph. D., Recorder of the Association. Printed for the Association. For sale by William J. Dornan, Philadelphia. 1897.

This volume contains the papers read before the association at the meeting held May 4–6, 1897, at Washington. It contains the proceedings at the unveiling of the bronze statue of the late professor Samuel D. Gross, M. D., erected at Washington by the American Surgical Association and the alumni association of Jefferson Medical College. These embrace the following items: frontispiece, half-tone engraving of the statue; history of the statue; order of exercises; address at the unveiling by Claudius Henry Mastin, M. D., A. M., LL. D., of Mobile; address on receiving the statue on behalf of the general government by Brigadier-general George M. Sternberg, M. D., LL. D., surgeon-general U. S. Army; address by William W. Keen, M. D., LL. D., of Philadelphia. A pamphlet containing these proceedings has been issued and will be supplied by the printer, Mr. William J. Dornan, at ten cents each.

Other interesting papers in this volume are the presidential address by J. Collins Warren, of Boston, on The influence of anesthesia on surgery; The indications for, and the technique of hysterectomy, by John Homans; The Röntgen rays in surgery, by J. William White; The technique of intracranial surgery, by Louis McLane Tiffany; Symptoms and treatment of hepatic abscess, by George Ben Johnston, and a number of papers relating to the surgery of the abdominal and pelvic viscera, including the intestines.

This was one of the largest and most interesting volumes ever issued by the association.

---

The Year-Book of Treatment for 1898. A critical review for practitioners of medicine and surgery. Crown octavo, 488 pages. Philadelphia and New York: Lea Brothers & Co. 1898.

This annual aims to be an epitomé of the most important medical advances of the year preceding its issue. This being the fourteenth volume, ample testimony is thereby given of its popularity. It is well edited and its contributors are men of experience. Its twenty-five chapters are brimful of information that is condensed and easily accessible. We know of no annual that affords the reader as much for his money (cloth, $1.50) as does this one and the possessor of the fourteen volumes may be regarded as extremely fortunate.

The arrangement of the book is very systematic and it is admirably indexed, thus bringing its subject-matter within convenient and easy reference.

---

Elements of Latin. For students of medicine and pharmacy. By George D. Crothers, A. M., M. D., Teacher of Latin and Greek in the St. Joseph (Mo.) High School; and Hiram H. Bice, A. M., Instructor in Latin and Greek in the Boys' High School of New York City, pp. xii.—242. Philadelphia, New York, Chicago: The F. A. Davis Co., Publishers.

An excellent book for those for whom it is designed. It simplifies very much the study of Latin for those unfamiliar with that language.

